# Bloody Mysteries of Ribosomes

**DOI:** 10.1097/HS9.0000000000000095

**Published:** 2018-07-09

**Authors:** Sergey O. Sulima, Kim De Keersmaecker

**Affiliations:** Department of Oncology, KU Leuven and Leuven Cancer Institute, Leuven, Belgium

Eukaryotic cells contain many thousands to millions of ribosomes distributed throughout the cytoplasm and attached to the endoplasmic reticulum. Capping the central dogma of biology, these ribosomes ensure faithful conversion of genomic information captured in mRNA into functional proteins. Impaired ribosome assembly and/or function stemming from mutations in one of the hundreds known ribosome assembly factors or 1 of the 80 ribosomal proteins is the underlying cause of the congenital disorders referred to as ribosomopathies.^1^ While these disorders display a broad spectrum of phenotypic defects such as growth retardation, pancreatic insufficiency, and neurocognitive impairment, there is a strong over-representation of hematopoietic deficiencies. For example, anemia and bone marrow failure are observed across different ribosomopathies—each caused by mutations in a different ribosomal protein or assembly factor—including Diamond-Blackfan anemia (DBA), Dyskeratosis Congenita, Shwachman-Diamond Syndrome (SDS), Myelodysplastic syndrome with Chromosome 5q Deletion, and Cartilage Hair Hypoplasia. Why is hematopoietic dysfunction a common nexus for an otherwise extremely diverse and unique set of phenotypic defects of ribosomopathies? This question is as important as it is puzzling. What causes mutations in the biochemical machine found in every single cell of the body, and with an equally essential role in every tissue, to have a more profound effect on hematopoietic cells? Moreover, in addition to the congenital disorders, a number of recent reports also described somatic ribosomal mutations, primarily found in hematologic cancers such as T-cell acute lymphoblastic leukemia, chronic lymphocytic leukemia, and multiple myeloma.^2^ Hematopoietic lineages thus appear to be a preferential target for both congenital and somatic ribosomal mutations. Several new exciting studies have shed some light on the “hema-centrism” of ribosomal defects, some of which are discussed below. Broad insights into this tissue-specificity paradox can also be appreciated in recent excellent reviews.^3,4^

Ribosomopathic mutations result in haploinsufficiency of key ribosome assembly factors and core ribosomal proteins, leading to defects in ribosome assembly and reduction of the cellular ribosome pool. This leaves fewer ribosomes available for cells to produce required proteins, which can lead to proliferation defects. Additionally, disrupted ribosome assembly increases the availability of free ribosomal proteins, which can activate TP53 and further augment the hypo-proliferative phenotypes. It is tempting to speculate that lower levels of functional ribosomes affect rapidly dividing cells, such as hematopoietic progenitors, more substantially. Indeed, the highest known rates of protein synthesis occur in progenitors undergoing erythroid lineage commitment.^5^ Other tissues that have high protein synthesis rates in the body, including the liver, gastrointestinal tract, muscle, and skin, are, however, not classically affected in ribosomopathies. Therefore, in addition to large protein synthesis requirements, other unique features of hematopoietic cells likely contribute to their sensitivity to ribosome defects. To this end, Mills et al delineated an erythroid-specific pathway with strong implications on ribosome function.^6^ After reaching a termination codon, ribosomes need to be recycled back to the beginning of the message for another round of translation or to begin translation of another message. Occasionally, ribosomes become trapped on an mRNA and can be freed by ribosome rescue factors. The authors found that the levels of the canonical ribosome recycling factor ABCE1 and rescue factor PELOTA are strongly and selectively reduced in reticulocytes after terminal differentiation. Resulting inefficient ribosome recycling might synergize with a shortage of ribosomes in reticulocytes to create an additive sensitivity to an imbalance in ribosome homeostasis. Furthermore, several studies demonstrated the essential role of ribosome levels on hematopoietic differentiation. It was previously shown that DBA-associated ribosomal protein defects promote defective translation of erythroid regulators of hematopoiesis BAG1, CSDE1, and GATA1.^7,8^ In a more recent follow-up,^9^ DBA-associated ribosomal protein lesions were observed to cause a reduction in ribosome levels, which in turn reduced translation of transcripts that are normally translated most rapidly and efficiently—those containing short and unstructured 5′ untranslated regions (UTRs)—including GATA1. Conversely, transcripts of the regulators of other hematopoietic lineages were found to contain long and structured 5′ UTRs, offering protection from the translational alterations of DBA-mutant ribosomes. The authors propose a model in which reduction of GATA1-specific translation in ribosome-mutant DBA impairs erythroid lineage commitment, ultimately causing erythroid deficiency. Similarly, deficiency for the ribosome assembly factor SBDS in SDS causes defective translation re-initiation on C/EBPα and C/EBPβ mRNAs,^10^ which might explain the neutropenia in SDS as these factors are essential for neutrophil proliferation and differentiation.

Whereas the defects described above may provide an explanation for defective erythropoiesis (DBA) and neutropenia (SDS), potential explanations regarding the disproportionate effect of ribosomal protein mutations on other hematopoietic lineages may emerge from the new provocative concept of “specialized ribosomes.” Because of their central role in all of life, ribosomes were historically assumed to be structurally and functionally invariable in every cell of an organism. Recent studies are challenging this notion. For example, a quarter of human ribosomal proteins were found to exhibit tissue-specific expression, with primary hematopoietic cells displaying the most complex expression patterns.^11^ Two recent papers by the group of Maria Barna lend additional support to this evolving idea by demonstrating that ribosomes can display heterogeneity both at the level of core ribosomal proteins^12^ and proteins interacting with ribosomes^13^ in murine embryonic stem cells. The authors identified populations of ribosomes with altered ribosomal protein composition, tasked for translation of specific mRNA subsets.^12^ Specifically, translation of classes of mRNAs containing strong secondary structures in the 5′ UTRs—such as Internal Ribosomal Entry Site elements—were found to depend on ribosomes with particular ribosomal protein composition. This study also indicates that ribosomal proteins found mutated in ribosomopathies like DBA are substoichiometric, and may demarcate ribosomes with specialized functions. This provides another example of how haploinsufficiencies of specific ribosomal proteins particularly perturb the translation of specific mRNAs, which may disproportionately result in hematopoietic dysfunction. In another approach, the authors focused on a complementary aspect of ribosomal function—interaction with extra-ribosomal factors.^13^ The large surface area of ribosomes, which increases along with organismal complexity, enables potential interactions with a variety of *trans*-acting factors. The complete identification of such factors illuminated for the first time a map of the “ribo-interactome.” This revealed several hundred ribosome-associated proteins belonging to a wide spectrum of functional categories including ribosome function and modification and, more surprisingly, cell cycle, cell redox homeostasis, and metabolism. Moreover, different subcellular pools of ribosomes displayed different ribosome-associated proteins, suggesting that ribosomal function may be *trans*-modulated at distinct cellular environments. This study also established a metazoan-specific post-translational modification termed ufmylation, in which UFM1 proteins are conjugated to particular ribosomal proteins. While the precise roles of this modification remain to be determined, the available knockout mouse models for the enzymes of the ufmylation cascade show defects in erythrocyte differentiation and result in embryonic lethality,^14^ similar to the defects arising from haploinsufficiency of some ribosomal proteins.

**Figure 1:**
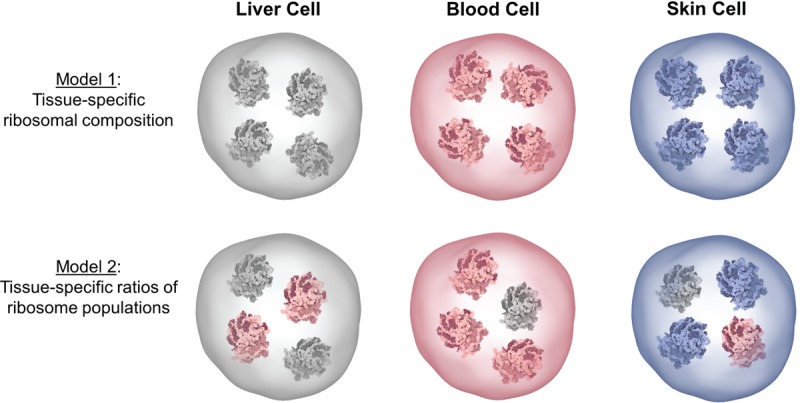
Models of ribosome heterogeneity between tissues.

These studies highlight the sensitivity of hematopoietic cells to defects in protein production, while underscoring the increasingly more heterogeneous, dynamic, and malleable nature of ribosomes. Adaptable ribosomes whose function can be modulated by differences in ribosomal composition or *cis*-and *trans*-regulation are rapidly replacing the outdated concept of “the” ribosome. While still in its infancy, this recent rise of the ribosomal repertoire might be a key to solving the tissue-specificity paradox of ribosome-mutant diseases. Evidence is accumulating that hematopoietic tissues, as well as other types of tissues, contain structurally and functionally unique ribosomes (Fig. 1, Model 1). In addition, it was recently proposed that cells contain a large diversity of heterogeneous ribosome populations, each with slightly fine-tuned, specialized function (Fig. 1, Model 2).^15^ The precise balance of these ribosome species varies with the environment (eg, tissues) and gets disrupted in disease states. We imagine that depletion of certain ribosome species due to decreased expression of a ribosomal protein, or introduction of new ribosome species due to missense mutations, would shift the overall balance of cellular ribosome variants, and hence the proportion of ribosomes with particular, optimized functions. Divergent function and regulation of the ribosomal molecular machine can be compared to the combustion engine: while the fundamental underlying mechanism remains the same, many subtle variations of the engine exist which are fine-tuned to a diversity of applications. The specialized engine driving translation in hematopoietic lineages might make these cells more vulnerable to faults in engine composition, activation, and maintenance.
